# Diminished Neurogenic Femoral Artery Vasoconstrictor Response in a Zucker Obese Rat Model: Differential Regulation of NOS and COX Derivatives

**DOI:** 10.1371/journal.pone.0106372

**Published:** 2014-09-12

**Authors:** Ana Cristina Martínez, Medardo Hernández, Susana Novella, María Pilar Martínez, Rosa María Pagán, Carlos Hermenegildo, Albino García-Sacristán, Dolores Prieto, Sara Benedito

**Affiliations:** 1 Departamento de Fisiología, Facultad de Farmacia, Universidad Complutense de Madrid, Madrid, Spain; 2 Departamento de Fisiología, Facultad de Medicina, Universidad de Valencia, Valencia, Spain; 3 Departamento de Anatomía y Anatomía Patológica Comparadas, Facultad de Veterinaria, Universidad Complutense de Madrid, Madrid, Spain; University of Southampton, United Kingdom

## Abstract

**Objective:**

Peripheral arterial disease is one of the macrovascular complications of type 2 diabetes mellitus. This study addresses femoral artery regulation in a prediabetic model of obese Zucker rats (OZR) by examining cross-talk between endothelial and neural factors.

**Methods and Results:**

Arterial preparations from lean (LZR) and OZR were subjected to electrical field stimulation (EFS) on basal tone. Nitric oxide synthase (NOS) and cyclooxygenase (COX) isoform expression patterns were determined by immunohistochemical labelling and Western blotting. Results indicate significantly reduced noradrenergic contractions in preparations from OZR compared with those of LZR. Functional inhibition of endothelial NOS (eNOS) indicated a predominant role of this isoform in LZR and its modified activity in OZR. Neural (nNOS) and inducible NOS (iNOS) were activated and their expression was higher in femoral arteries from OZR. Neurotransmission modulated by large-conductance Ca^2+^-activated (BK_Ca_) or voltage-dependent (K_V_) K^+^ channels did not seem compromised in the obese animals. Endothelial COX-1 and COX-2 were expressed in LZR and an additional adventitial location of COX-2 was also observed in OZR, explaining the higher COX-2 protein levels detected in this group. Prostanoids derived from both isoforms helped maintain vasoconstriction in LZR while in OZR only COX-2 was active. Superoxide anion inhibition reduced contractions in endothelium-intact arteries from OZR.

**Conclusions:**

Endothelial dysfunction led to reduced neurogenic vasoconstriction in femoral arteries from OZR. In a setting of obesity, NO-dependent nNOS and iNOS dilation activity could be an alternative mechanism to offset COX-2- and reactive oxygen species-mediated vasoconstriction, along with impaired endothelial NO relaxation.

## Introduction

Peripheral arterial disease (PAD), a clinical manifestation of atherosclerosis, is an independent risk factor of mortality and morbidity in patients with cardiovascular diseases. Among the complications of PAD, foot ulcers and gangrene may determine a need for lower extremity amputation, with the consequent loss of mobility for the patient. Patients with diabetes mellitus (DM) show a high prevalence of PAD and DM is today the leading cause of nontraumatic lower-limb amputation in developed countries [1].

The significant role played by factors such as obesity, hyperglycemia, hyperlipidemia, inflammation, and oxidative stress in the progression of diabetic complications has been well established [2], [3].

Several lines of evidence suggest that endothelial dysfunction could be a major contributor to both macro- and microangiopathy in DM patients and in animal models of diabetes [4], [5]. Deficiencies in nitric oxide (NO), endothelium-derived hyperpolarizing factor and prostacyclin will affect the balance between vasodilatation and vasoconstriction. The upregulated release of endothelium-derived contractile factors, such as prostanoids derived from cyclooxygenase (COX) [6], the production of vasoconstrictor peptides, such as endothelin [7] or of radical oxygen species (ROS) [8] are also hallmarks of endothelial dysfunction.

The obese Zucker rat (OZR), genetic model of obesity-related insulin resistance [9] used in this study lacks functional leptin receptors and features the characteristics obesity, hyperglycemia, hyperinsulinemia and hyperlipidemia. This model therefore closely mimics the clinical situation of a prediabetes state, in which there is an increased risk of chronic limb ischemia. As controls, we subjected lean Zucker rats (LZR) to the same experimental protocol.

According to prior findings for the penile and coronary arteries of OZR, this model shows vascular remodeling and endothelial dysfunction along with increased oxidative stress and the reduced bioavailability of endothelial vasodilators, such as NO and prostacyclin [10]–[14].

The aim of this study was to examine the OZR model to assess the possibility of impaired excitatory neurotransmission in the femoral artery, as the most common anatomic location of PAD. Also examined were the endothelial and neural components of the femoral artery’s neurogenic response and their responsible mechanisms.

## Methods

### Animal model

The investigation conformed to the European Union Guidelines for the Care (European Union Directive (2010/63/EU) and the Use of Laboratory Animals and all the experimental protocols were approved by the Institutional Animal Care and Use Committee of Madrid Complutense University.

Male LZR and OZR were purchased from Charles River Laboratories (Barcelona, Spain) at 8–10 weeks of age. Animals were housed at the Pharmacy School animal care facility, maintained under standard rat room conditions and fed on standard rat chow and tap water ad libitum, until they were used for study, at 17–18 weeks age.

Rats were sacrificed by cervical dislocation and exsanguination. The femoral arteries from LZR and OZR were removed, carefully cleaned and cut into 2 mm segments.

### Measurement of isometric force

Femoral artery segments were mounted isometrically in a wire microvascular myograph system (DMT, Aarhus, Denmark) using two 40 mm tungsten wires, for measuring generated force. The chambers were kept at 37°C and bubbled continuously with 95% O_2−_5% CO_2_ in physiological saline solution (PSS) to keep the pH at 7.4. The composition of PSS (mM) was: NaCl 119, KCl 4.7, CaCl_2_ 1.5, MgSO_4_ 1.2, NaHCO_3_ 25, glucose 11, KH_2_PO_4_ 1.2 and ethylenediaminetetraaceticacid (EDTA) 0.027.

The preparations were allowed to equilibrate for about 30 min in PSS and washed with (37°C) PSS at 15 min intervals. After this equilibration period, each ring was stretched at 1 min intervals to determine the relationship between passive wall tension and internal circumference (L). We also calculated the internal circumference, L_100,_ corresponding to a transmural pressure of 100 mmHg in a relaxed vessel. Subsequently, the internal circumference of the vessels was set at L_1_, where L_1_ = 0.9 × L_100_ since force development is close to maximal at this internal circumference. Isometric tension was recorded and displayed using a Powerlab data system supplied with Chart v5.5 (AD Instruments, Hastings, U.K.). At the beginning of each experiment the preparations were stimulated once with a potassium-enriched solution (K-PSS) to check the contractile ability of the arteries. To prepare K-PSS, the NaCl in PSS was replaced with KCl on an equimolar basis.

To study the role of endothelium, the luminal surface was gently rubbed with a human hair, and successful removal of endothelium was confirmed by the inability of acetylcholine (1–10 microM) to relax segments precontracted with serotonin (5-HT, 0.3 microM).

Electrical field stimulation (EFS) was performed using two 2×2 mm platinum electrodes (DMT, Aarhus, Denmark) secured in plastic mounting heads on either side of the preparations, approximately 1 mm from the vessel wall. The electrodes were connected to an electrical stimulator CS-20 (Cibertec, Barcelona, Spain).

To obtain reproducible contractions to EFS, neuronal noradrenalin re-uptake and β-adrenoceptors were blocked by incubating the arteries for 30 min before to stimulation with, cocaine (3 microM) and propranolol (3 microM) respectively. Two consecutive reproducible frequency-response curves (0.5–32 Hz, 20 s trains, 0.2 ms pulses, 65 mA) were obtained in preparations under resting tension [15]. To study the mechanisms underlying EFS- induced contractile responses, different antagonists, inhibitors and blockers were added to the organ bath 30 min before conducting the second frequency- response curves.

In preliminary experiments, EFS was applied in the presence of a blocker of neuronal voltage-gated Na^+^ channels, tetrodotoxin (1 microM), to assess the neurogenic character of the EFS- elicited responses.

To study the nature of EFS- induced contractile responses the arterial segments were treated with the adrenergic neurotransmission blocker, guanethidine (10 microM), the alpha-adrenoceptor antagonist, phentolamine (10 microM), the selective alpha_1_-adrenoceptor antagonist, prazosin (1 microM). To evaluate the role of NO, preparations were incubated with N^omega^-nitro-L-arginine (L-NOARG, 100 microM) to not selectively inhibit NO synthase (NOS) or with N^omega^-propyl-L-arginine (3 microM) to inhibit neuronal NOS (nNOS) or with 1400 W (10 microM) to inhibit inducible NOS (iNOS). The type of potassium channel was determined using the non-selective Ca^2+^-activated K^+^ (K_Ca_) channel blocker, tetraethylammonium (TEA, 1 mM); specific blockers such as the blockers of large- (BK_Ca_), iberiotoxin (0.1 microM); intermediate- (IK_Ca_), TRAM-34 (3 microM); or small- conductance K_Ca_ channels (SK_Ca_) apamin (0.3 microM); of voltage-dependent K^+^ channels (K_V_), 4-aminopyridine (100 microM); of ATP-dependent K^+^ channels (K_ATP_), glibenclamide (1 microM); or inward rectifier K^+^ channels (K_ir_), barium chloride (BaCl_2_, 30 microM). Ouabain (10 microM), a Na^+^-K^+^ ATPase pump inhibitor was also used. Contribution of prostanoids to EFS- induced responses was investigated by using the non-selective cyclooxygenase (COX) inhibitor, indomethacin (1 microM) and selective COX-1 and COX-2 inhibitors, SC-560 (1 microM) and NS-398 (1 microM) respectively. Involvement of reactive oxygen species, NADP(H)-oxidase and endothelin receptors in the contractile responses was analysed by adding to the organ bath superoxide dismutase (SOD, 150 U/mL) or apocynin (100 microM) and bosentan (10 microM), respectively.

### Immunohistochemistry

Femoral artery segments were fixed by immersing in 4% paraformaldehyde prepared in 0.1 M sodium phosphate buffer (PB), pH 7.4 at 4°C for 3–4 h, and then placed in a cryoprotective phosphate buffer solution containing 30% sucrose for 24 h at 4°C. The tissue was embedded and frozen in OCT compound (Tissue-Tek. Sakura Finetek, Europe B.V.), and stored at −80°C. Transversal sections 5 microm thick were obtained by means of a cryostat.

For immunohistochemistry using avidin–biotin–peroxidase complex procedures [16], the tissue sections were immersed in a mixture of 1% H_2_O_2_ and 90% methanol in distilled water and then washed in PB (3×10 min). Specimens were preincubated for 3 h in 10% normal goat serum in PB containing 0.3% Triton X-100 to detect endothelial, neuronal and inducible NOS, and in 5% bovine serum albumin and 10% normal goat serum in PB containing 0.3% triton X-100 to detect COX-1 and COX-2. Next, the sections were treated with rabbit anti-eNOS antibody (Chemicon International Inc.) diluted 1∶500 in PB, rabbit anti-nNOS (Chemicon International Inc.) diluted 1∶1000 in PB, rabbit anti-iNOS (Santa Cruz Biotechnology Inc.) diluted 1∶50 in PB and rabbit anti-COX-1 and COX-2 (Santa Cruz Biotechnology Inc) diluted 1∶50 in PB. Sections were then incubated for 2 h at room temperature with biotinylated anti-rabbit serum raised in goat (Chemicon International Inc., Phillipsburg, NJ, USA) diluted 1∶400. Once treated with the avidin–biotin complex (ABC, Vector) diluted 1∶100 for 90 min at room temperature, the resultant immunocomplex was visualized using 0.05% 3,3′-diaminobenzidine and 0.001% H_2_O_2_ in PB. No immunoreactivity could be detected in sections incubated in the absence of the primary antisera.

### Western blots

Femoral artery segments were homogenized (MagNA Lyser, Roche, IN, USA) in radioimmunoprecipitation assay (RIPA) buffer containing protease inhibitor cocktail tablets at the concentration supplied by the manufacturer (Complete Mini, Roche, IN, USA). Protein contents were determined using the BCA (bicinchoninic acid) Protein Assay Kit (Thermo Scientific Pierce, Madison, WI, USA) and equal amounts of whole vessel homogenates were electrophoresed on 7.5% SDS-polyacrylamide and transferred to polyvinylidene difluoride membranes. After blocking, the membranes were incubated overnight (4°C) with primary antibodies against eNOS (Santa Cruz Biotechnology Inc, Santa Cruz, CA, USA), iNOS (BD Transduction Laboratories, Madrid, Spain), nNOS (BD Transduction Laboratories, Madrid, Spain), COX-1 (Santa Cruz Biotechnology Inc., Santa Cruz, CA, USA) and COX-2 (Santa Cruz Biotechnology Inc, Santa Cruz, CA, USA). As positive controls were used cell lysate from mouse macrophages stimulated with 10 ng/mL interferon gamma (IFNgamma) and 1 microg/mL lipopolysaccharide (LPS), for iNOS (BD Transduction Laboratories, Madrid, Spain); rat cerebrum lysate for nNOS (BD Transduction Laboratories, Madrid, Spain); cell lysates from human umbilical vein endothelial cells (HUVEC) for eNOS and COX-1; and cell lysate from HUVEC treated with 1 microg/mL LPS for COX-2. As internal control of the amount of protein, beta-actin (Sigma–Aldrich, St. Louis, MO, USA) was used. After incubation with secondary antirabbit or anti-mouse antibodies as appropriate, antibody binding was assessed by enhanced chemiluminescence (Thermo Scientific Pierce ECL Western Blotting Substrate, Madison, WI, USA). Blots were digitalized using a Gelprinter Plus (TDI, Madrid, Spain) and the densities of spots assessed using Image Gauge 4.0 software (Fujifilm Science Lab, Madrid, Spain).

### Drugs and solutions

The following drugs were used: acetylcholine, 4-aminopyridine, apamin, 4′-hydroxy-3′-methoxyacetophenone (apocynin), barium chloride, cocaine, glibenclamide, guanethidine, 5-HT, iberiotoxin, indomethacin, L-NOARG, N^omega^-propyl-L-arginine, phentolamine, ouabain, prazosin, propranolol, SOD, tetrodotoxin, TEA and 1-(2-Chlorophenyl) diphenylmethyl- 1H-pyrazole (TRAM-34) (Sigma Chemical Co, St Louis, MO, USA). NS-398, SC-560 and 1400 W were purchased from Tocris Cookson (Bristol, UK). Bosentan was a gift from F. Hoffman-La Roche Laboratories (Basel, Switzerland).

All drugs were added in volumes not exceeding 0.3% of the organ baths to reach the final required concentration. They were dissolved in distilled water with the exception of indomethacin which was prepared in ethanol (96%), glibenclamide, SC-560 and NS-398 which required dimethylsulphoxide (10%). Preliminary experiments revealed no effects of the solvent used on the contractility of the preparations. Stock solutions were prepared and stored at −20°C and fresh solutions were prepared daily.

### Data calculations and statistics

The mechanical responses of the arteries were expressed as active wall tension (N·m^−1^). Contractions are either expressed as tension or as percentage of contraction induced by K-PSS. Results are expressed as means ± S.E.M. of n rats. Statistical differences were analyzed using one-way ANOVA or paired or unpaired Student’s *t*-test when appropriate. Probability levels less than 5% were considered significant (*P*<0.05). All calculations were made using a standard software package (Prism 4.0, GraphPad San Diego, CA).

## Results

### General parameters

The OZR were significantly heavier (474.3±4.8 g versus 370.3±4.7 g; *P<0.001*, n = 40) than LZR (both strains 17–18 weeks of age). We previously reported that at this age animals in the obese group show mild hyperglycemia, hyperinsulinemia and dyslipidemia with elevated total cholesterol and triglyceride levels [11]. Normalized vessel internal lumen diameters, L_1_, were similar in the OZR (443.6±7.8 microm) and lean animals (435.3±10.8 microm; n = 40). Also, reactivity to depolarization with K-PSS was comparable in both groups of arteries (LZR, 10.6±0.7 N·m^−1^; OZR, 11.2±0.9 N·m^−1^; n = 40), suggesting the unaltered contractility of arterial smooth muscle.

### Reduced neurogenic vasoconstriction in femoral arteries from OZR

**Figure 1:**
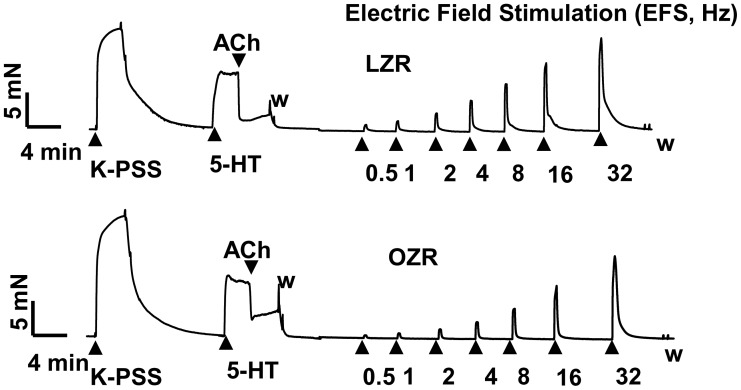
Decreased neurogenic vasoconstrictor response from obese Zucker rat femoral artery. Representative tracings showing the contractile effect evoked by electrical field stimulation (EFS) in femoral artery from LZR and OZR. ACh, acetylcholine (1 microM); 5-HT, serotonin (0.3 microM); W, washout. Vertical bar indicates increase in force (mN), and horizontal bar indicates time (min).

**Figure 2:**
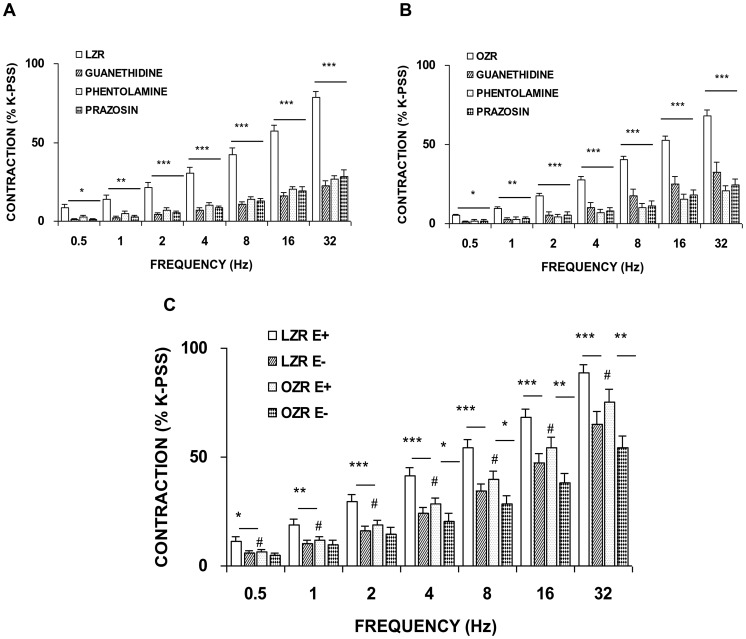
Role of the endothelium in adrenergic responses induced by EFS. Neurogenic contractions were obtained in the absence and presence of guanethidine (10 microM), phentolamine (10 microM) and prazosin (1 microM) in femoral artery from LZR (A) and OZR (B). Data are means ± S.E.M. for values obtained in 7–9 animals. (C) Neurogenic contractions were obtained in femoral artery with endothelium (E+) and without endothelium (E−) from LZR and OZR. Data are means ± S.E.M. for values obtained in 15–20 animals. *P<0.05; **P<0.01; ***P<0.001 significance compared to endothelium-intact segments; # *P*<0.05 obese *versus* lean.

EFS-evoked frequency-dependent contractions were of a lesser magnitude in femoral arteries isolated from OZR than LZR at all frequencies examined (Figure 1). EFS-induced contractions were abolished by the neuronal voltage-gated Na^+^ channel blocker, tetrodotoxin (1 microM), and markedly reduced by guanethidine (10 microM), a noradrenergic neurotransmission inhibitor, phentolamine (10 microM), an alpha-adrenoceptor antagonist, and prazosin (1 microM), a selective alpha_1_ -adrenoceptor antagonist (Figure 2A and B). Similarly, alpha_1_-adrenergic receptor blockade potently inhibited the contraction induced by EFS in rings devoid of endothelium (data not shown). These results indicate that noradrenaline is the main contributor to the neurogenic contraction, via alpha_1_ receptor activation.

Endothelial cell removal reduced contractions induced by EFS compared to endothelium-intact preparations in both strains, though to a greater extent in LZR (Figure 2C). Having ruled out the presence of endothelial alpha_1_ adrenoceptors, our results indicate that endothelial denudation has differential effects on excitatory neurotransmission in arteries obtained from both experimental groups (Figure 2C).

### Expression and function changes induced in the NOS isoforms involved in neurogenic vasoconstriction in OZR

**Figure 3:**
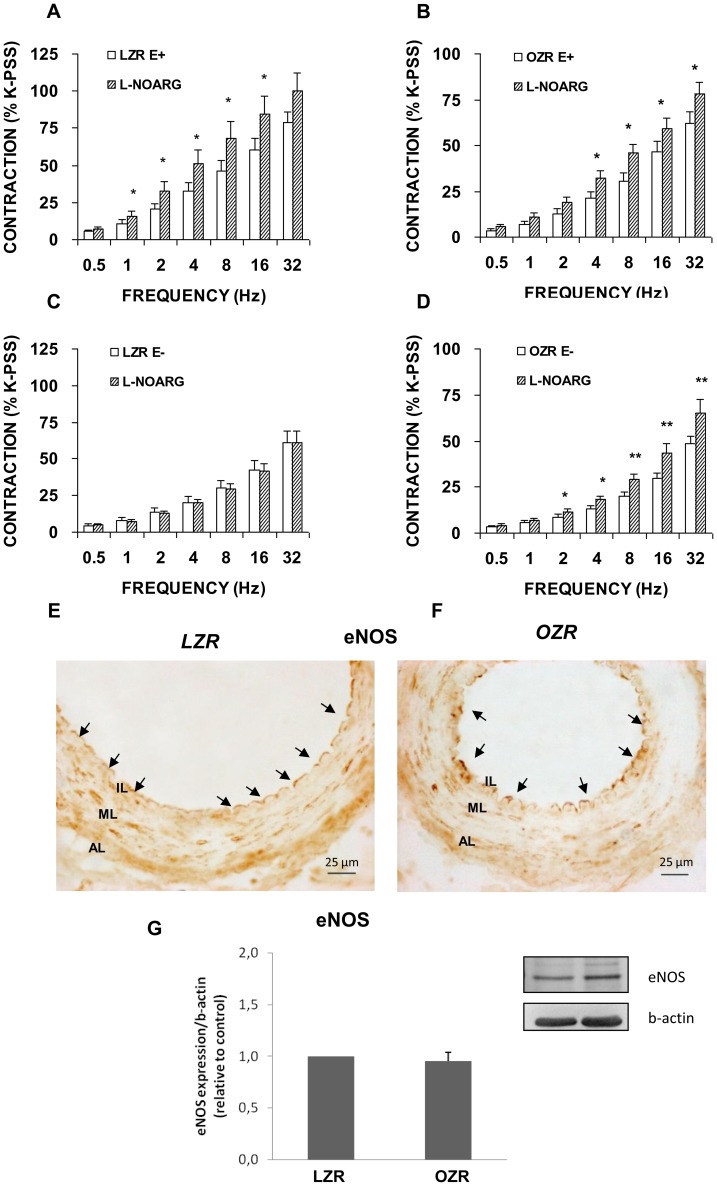
Role of eNOS in contractile responses induced by EFS. Neurogenic contractions were obtained in the absence and presence of a NOS inhibitor, L-NOARG (100 microM) in femoral artery with endothelium (E+) from LZR (A) and OZR (B) and without endothelium (E−) from LZR (C) and OZR (D). Data are means ± S.E.M. for values obtained in 7–8 animals. *P<0.05; **P<0.01 *versus* L-NOARG-treated. Representative original pictures of cross-sections of femoral artery from LZR (E) and OZR (F) showing immunoreactivity for eNOS in the endothelial cell layer (arrows). Note that localization of eNOS immunoreaction was similar in LZR and OZR. (G) A representative immunoblotting image and relative levels assessed by densitometry of eNOS protein expression in femoral artery from LZR and OZR are presented. Data are means ± S.E.M. for values obtained in 6 animals.

In arteries with an intact endothelium, L-NOARG (100 microM) potentiated the contractile responses of the arterial rings to sympathetic neurogenic stimulation in LZR (Figure 3A), but did not modify EFS contractile responses in denuded segments from the same group (Figure 3C). Conversely, in arterial segments from the OZR group, the contraction induced by EFS was enhanced in both intact and denuded preparations when NOS activity was inhibited (Figure 3B and D). Immunoreactivity for eNOS in cross sections of femoral arteries revealed no differences in the presence and distribution of this constitutive NOS isoform in the endothelium of femoral arteries in OZR (Figure 3F) compared with LZR (Figure 3E). Western blotting of eNOS protein confirmed no significant differences in the levels of expression between the LZR and OZR groups (Figure 3G). The expression of beta-actin was not significantly different between LZR and OZR.

**Figure 4:**
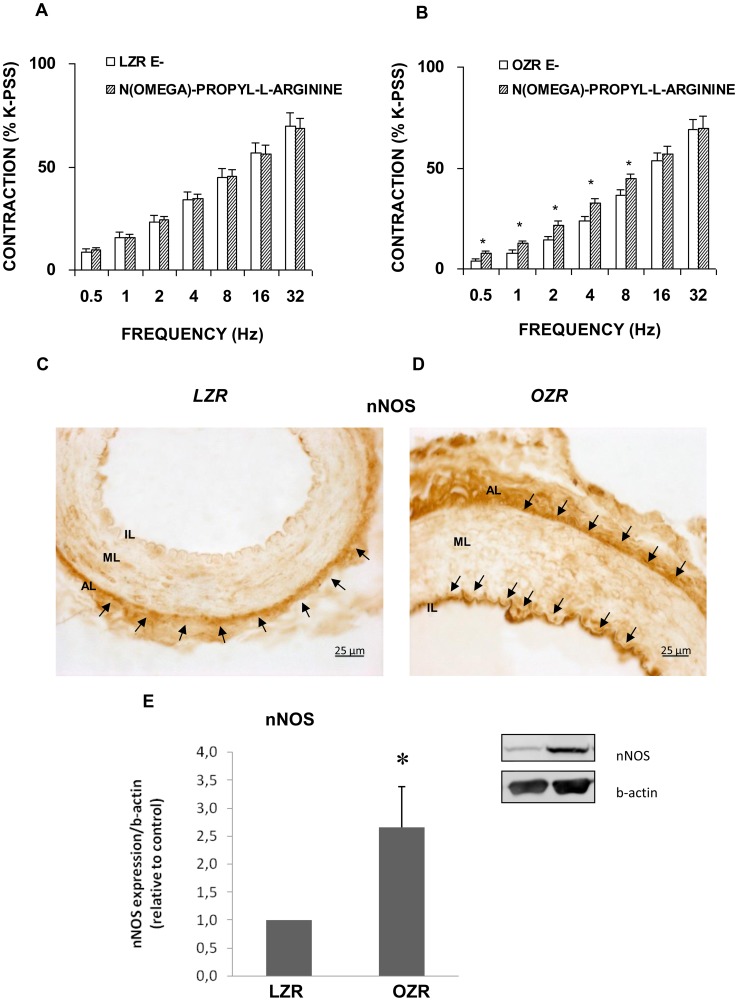
Role of nNOS in contractile responses induced by EFS. Neurogenic contractions were obtained in the absence and presence of selective neural NOS inhibitor, N^omega^-propyl-L-arginine (3 microM) in femoral artery without endothelium (E−) from LZR (A) and OZR (B). Data are means ± S.E.M. for values obtained in 7–8 animals. *P<0.05. Representative original pictures of cross-sections of femoral artery from LZR (C) and OZR (D) showing immunoreactivity for nNOS between the muscle and adventitia layer of the femoral arteries (arrows). Note that nNOS inmunoreaction was also localized in the endothelial cell layer of the femoral artery from OZR. (E) A representative immunoblotting image and relative levels assessed by densitometry of nNOS protein expression in femoral artery from LZR and OZR are presented. Data are means ± S.E.M. for values obtained in 6 animals. *P<0.05.

The selective nNOS inhibitor, N^omega^-propyl-L-arginine (3 microM), significantly enhanced vasoconstriction in endothelium-free femoral arteries in OZR (Figure 4B) but did not modify responses in LZR (Figure 4A). Arterial cross sections immunolabelled with an antibody against nNOS revealed higher nNOS immunoreactivity in OZR (Figure 4D) than LZR (Figure 4C). In LZR, nNOS expression was observed only within the adventitial perivascular nerves while in OZR this expression was also detected in the endothelial lining of the femoral artery (Figure 4D). Western blots supported the increased expression of nNOS in the arteries harvested from OZR (Figure 4E).

**Figure 5:**
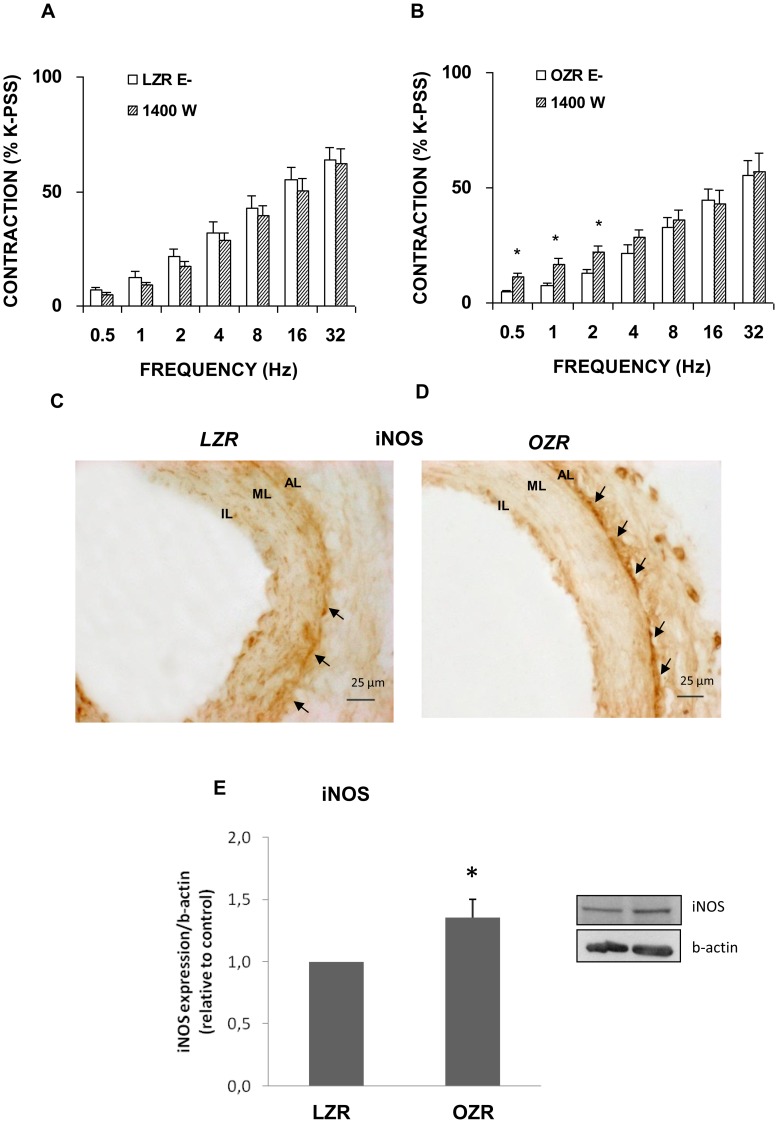
Role of iNOS in contractile responses induced by EFS. Neurogenic contractions were obtained in the absence and presence of selective inducible NOS inhibitor, 1400 W (10 microM) in femoral artery without endothelium (E−) from LZR (A) and OZR (B). Data are means ± S.E.M. for values obtained in 7–9 animals. *P<0.05. Representative original pictures of cross-sections of femoral artery from LZR (C) and OZR (D) showing immunoreactivity for iNOS between the smooth muscle and adventitia layer of the femoral arteries (arrows). Note that iNOS inmunoreaction was more intense in the femoral arteries from OZR. (E) A representative immunoblotting image and relative levels assessed by densitometry of iNOS protein expression in femoral artery from LZR and OZR are presented. Data are means ± S.E.M. for values obtained in 7–8 animals. *P<0.05.

Exposure of the denuded femoral rings to the iNOS selective inhibitor 1400 W (10 microM) led to increased vasoconstriction responses only in OZR, as also observed for nNOS (Figure 5A and B). Immunoreactivity against iNOS, more intense in femoral arteries from OZR, was observed between the smooth muscle and adventitia layers (Figure 5C and D). Also, we detected a significant increase in iNOS protein expression in femoral arteries from OZR by Western blotting (Figure 5E).

These observations suggest the induction of nNOS and iNOS activities in OZR in response to the loss of eNOS activity.

### Modulation of K^+^ channels of neurogenic vasoconstriction is unmodified in OZR

**Figure 6:**
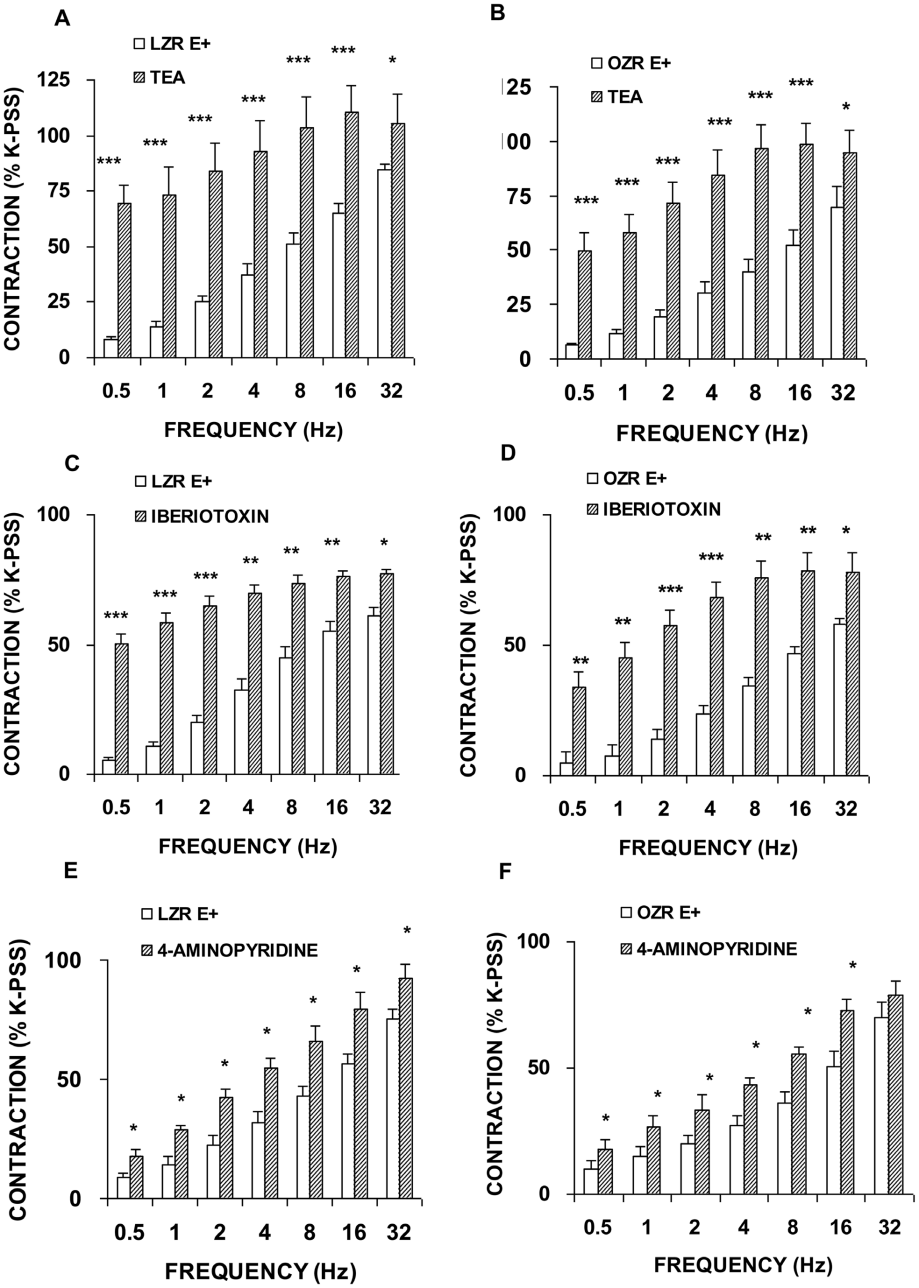
Preserved function of K^+^ channels in responses induced by EFS. Neurogenic contractions were obtained in femoral artery with endothelium (E+) in the absence and presence of TEA (1 mM), a non-selective K_Ca_ blocker from LZR (A) and OZR (B), in the absence and the presence of the BK_Ca_ blocker, iberiotoxin (0.1 microM) from LZR (C) and OZR (D) and in the absence and presence of a K_V_ blocker, 4-amynopiridine (100 microM) from LZR (E) and OZR (F). Data are means ± S.E.M. for values obtained in 6–7 animals. *P<0.05; **P<0.01; ***P<0.001.

TEA (1 mM), a non-selective blocker of K_Ca_ channels (Figure 6A and B), and the selective blocker of BK_Ca_ iberiotoxin (0.1 microM) (Figure 6C and D) increased the contraction response to EFS in femoral arteries obtained from LZR and OZR. The extent of potentiation of adrenergic contractions induced by these blockers was comparable between both groups. The blocker of IK_Ca_ TRAM-34 (3 microM), or SK_Ca_ apamin (0.3 microM) had no effect on neurogenic responses in LZR and OZR arteries (data not shown). The specific blocker of K_V_, 4-amynopiridine (0.1 mM), enhanced EFS-evoked contractions in LZR and OZR femoral arteries (Figure 6E and F). The magnitude of this increase was similar in both experimental groups. Blockade of K_ATP_ with glibenclamide (1 microM), and of Kir with BaCl_2_ (30 microM) and a Na^+^-K^+^ ATPase pump inhibitor, ouabain (10 microM) did not affect the contractions elicited by EFS in arterial rings from either group. These results were not significantly different to those recorded in denuded arteries (data not shown).

Collectively, these observations suggest that the condition of obesity does not affect the modulatory role of neural vasoconstriction of K^+^ channels, K_Ca_ and Kv, which are functionally active in the femoral artery of the Zucker rat.

### Expression and function changes induced in the COX isoforms involved in neurogenic vasoconstriction in OZR

**Figure 7:**
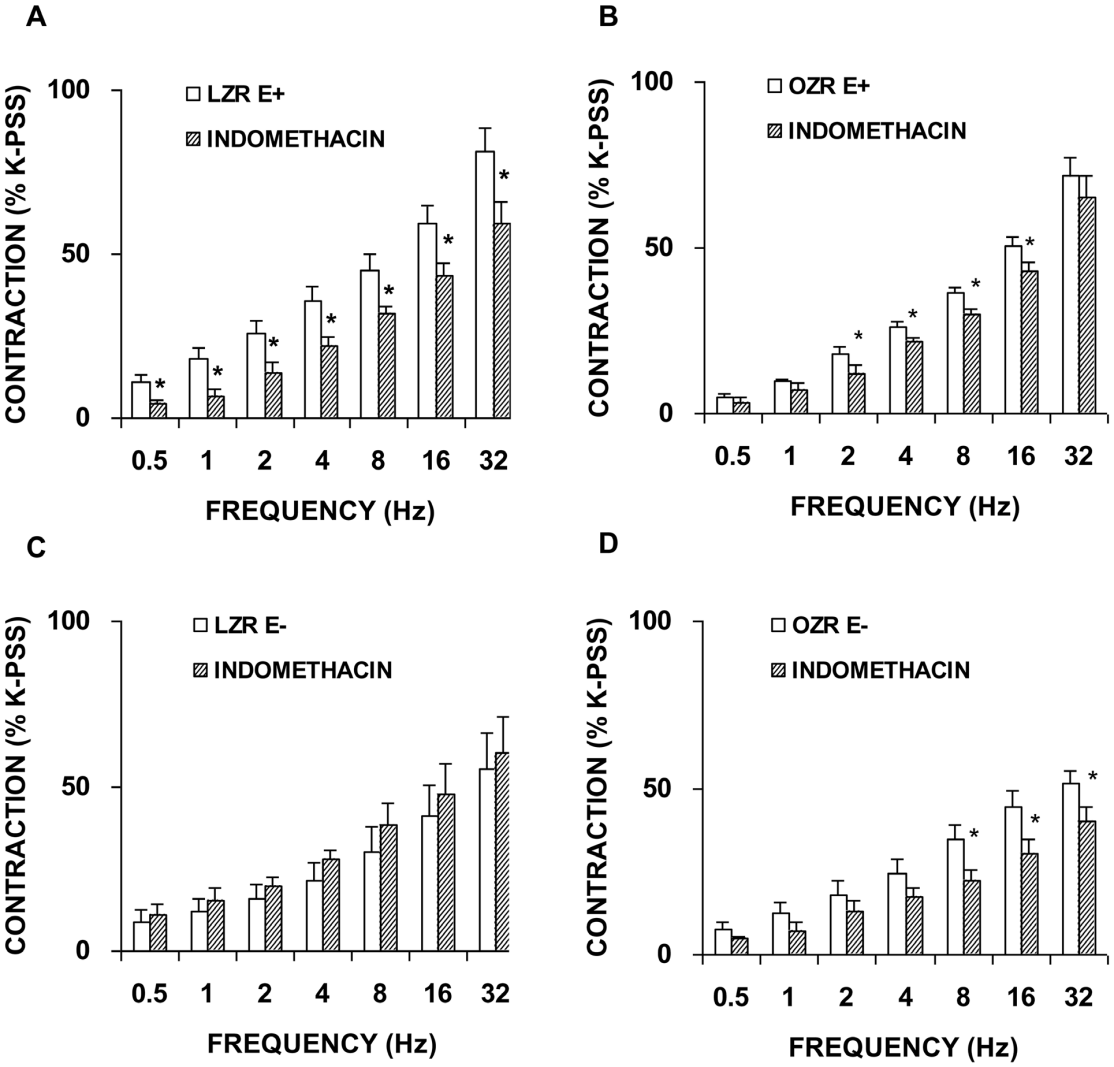
Role of COX in contractile responses induced by EFS. Neurogenic contractions were obtained in the absence and presence of a non-selective COX inhibitor, indomethacin (1 microM) in femoral artery with endothelium (E+) from LZR (A) and OZR (B), and without endothelium (E−) from LZR (C) and OZR (D). Data are means ± S.E.M. for values obtained in 6–7 animals. *P<0.05.

**Figure 8:**
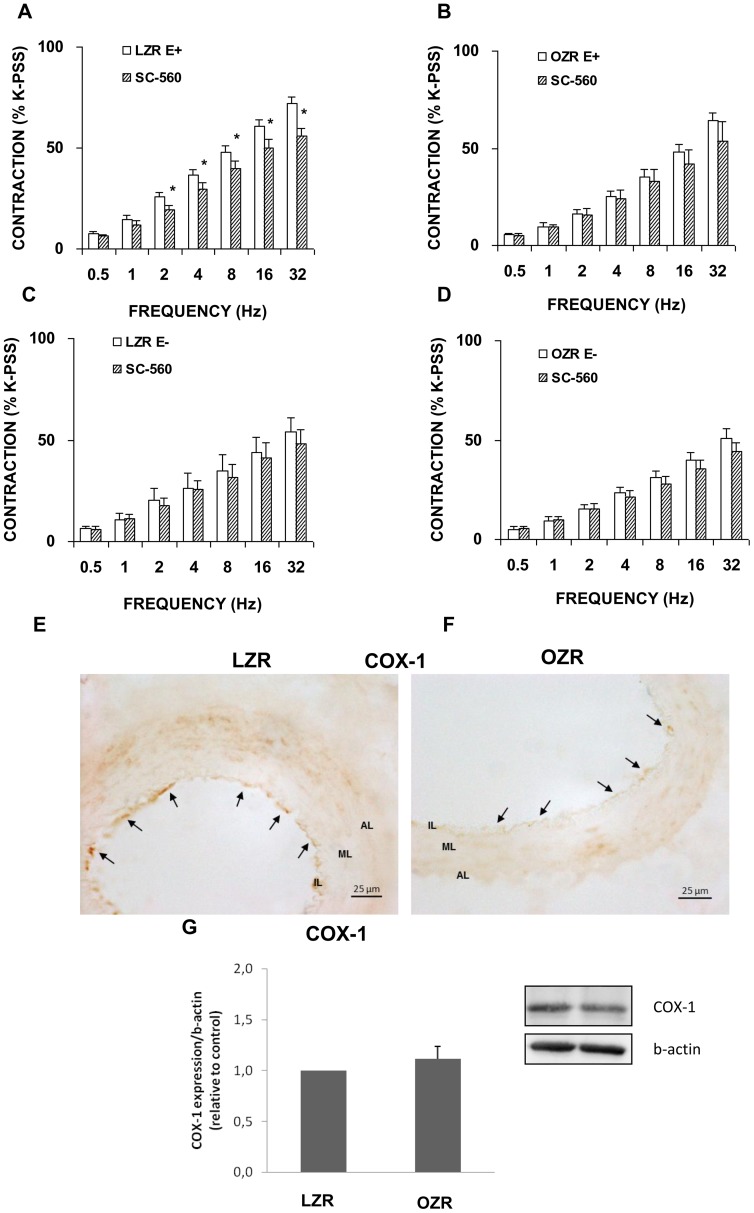
Role of COX-1 in contractile responses induced by EFS. Neurogenic contractions were obtained in the absence and presence of the selective COX-1, SC-560 (1 microM) in femoral artery with endothelium (E+) from LZR (A) and OZR (B) and without endothelium (E−) from LZR (C) and OZR (D). Data are means ± S.E.M. for values obtained in 6–7animals. *P<0.05 *versus* SC-560-treated. Representative original pictures of cross-sections of femoral artery from LZR (E) and OZR (F) showing immunoreactivity to COX-1 in endothelial layer (arrows). Note that localization of COX-1 immunoreaction was similar in LZR and OZR. (G) A representative immunoblotting image and relative levels assessed by densitometry of COX-1 protein expression in femoral artery from LZR and OZR are presented. Data are means ± S.E.M. for values obtained in 6 animals.

In the presence of the non-selective COX inhibitor, indomethacin (1 microM), a significant decrease in the contractile response to EFS was produced in arterial segments with an intact (Figure 7A) but not a denuded endothelium in the LZR group (Figure 7C). In femoral arteries from OZR, indomethacin significantly reduced adrenergic contractions both in segments with and without an endothelium (Figure 7B and D). To assess the specific COX isoform involved, we examined the effects of the selective COX-1 inhibitor, SC-560 (1 microM), and the selective COX-2 inhibitor, NS-398 (1 microM) on contractile responses to EFS. Treatment with SC-560 reproduced the effect of indomethacin in segments from LZR (Figure 8A and C), but in those from OZR, frequency-dependent responses were not inhibited, independently of the presence of endothelium (Figure 8B and D). Immunohistochemical staining showed the presence of the constitutive COX-1 isoform throughout the endothelial layer of femoral arteries in both strains (Figure 8E and F). Western blotting revealed a similar COX-1 expression pattern in segments from LZR and OZR (Figure 8G).

**Figure 9:**
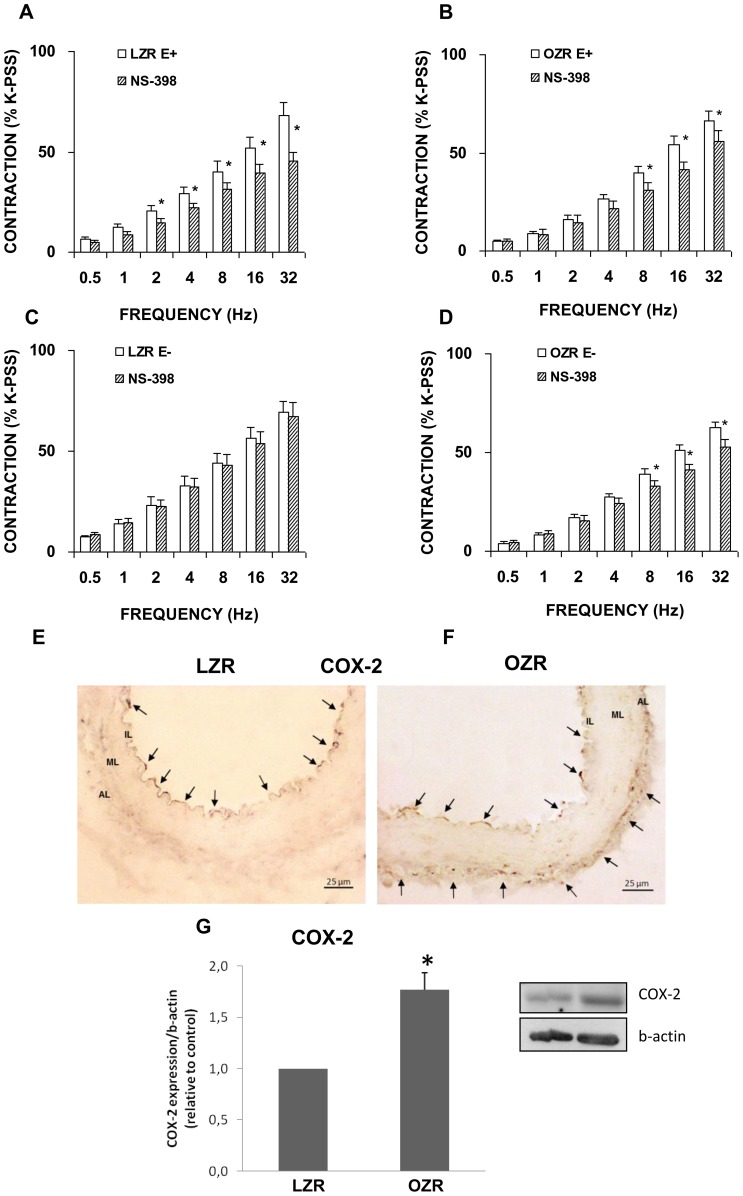
Role of COX-2 in contractile responses induced by EFS. Neurogenic contractions were obtained in the absence and presence of the selective COX-2, NS-398 (1 microM) in femoral artery with endothelium (E+) from LZR (A) and OZR (B) and without endothelium (E−) from LZR (C) and OZR (D). Data are means ± S.E.M. for values obtained in 6–7animals. *P<0.05 *versus* NS-398-treated. Representative original pictures of cross-sections of femoral artery from LZR (E) and OZR (F) showing immunoreactivity to COX-2 in endothelial layer (arrows). Note that additional COX-2 immunostaining was also found in the adventitial layer from OZR. (G) A representative immunoblotting image and relative levels assessed by densitometry of COX-2 protein expression in femoral artery from LZR and OZR are presented. Data are means ± S.E.M. for values obtained in 6 animals. *P<0.05.

Following COX-2 inhibition, EFS also gave rise to a reduced contraction response in LZR arteries with an intact endothelium (Figure 9A) whereas the contractile response was unaffected in matched arterial segments denuded of endothelium (Figure 9C). The contraction elicited by EFS in the presence of NS-398 was significantly diminished in OZR arteries with and without endothelium (Figure 9B and D). Femoral arterial sections labelled with antibody against COX-2, also showed that this isoform was primarily expressed in the arterial endothelial layer in LZR (Figure 9E) and the endothelial and adventitial layers in OZR (Figure 9F). Western blotting revealed augmented COX-2 protein contents in femoral arteries from OZR (Figure 9G). These findings seem to indicate that while the COX-1 isoform loses its functional activity, COX-2 is upregulated in arteries from obese animals.

### Other contractile factors involved in neurogenic vasoconstriction: key role of superoxide anions in OZR

**Figure 10:**
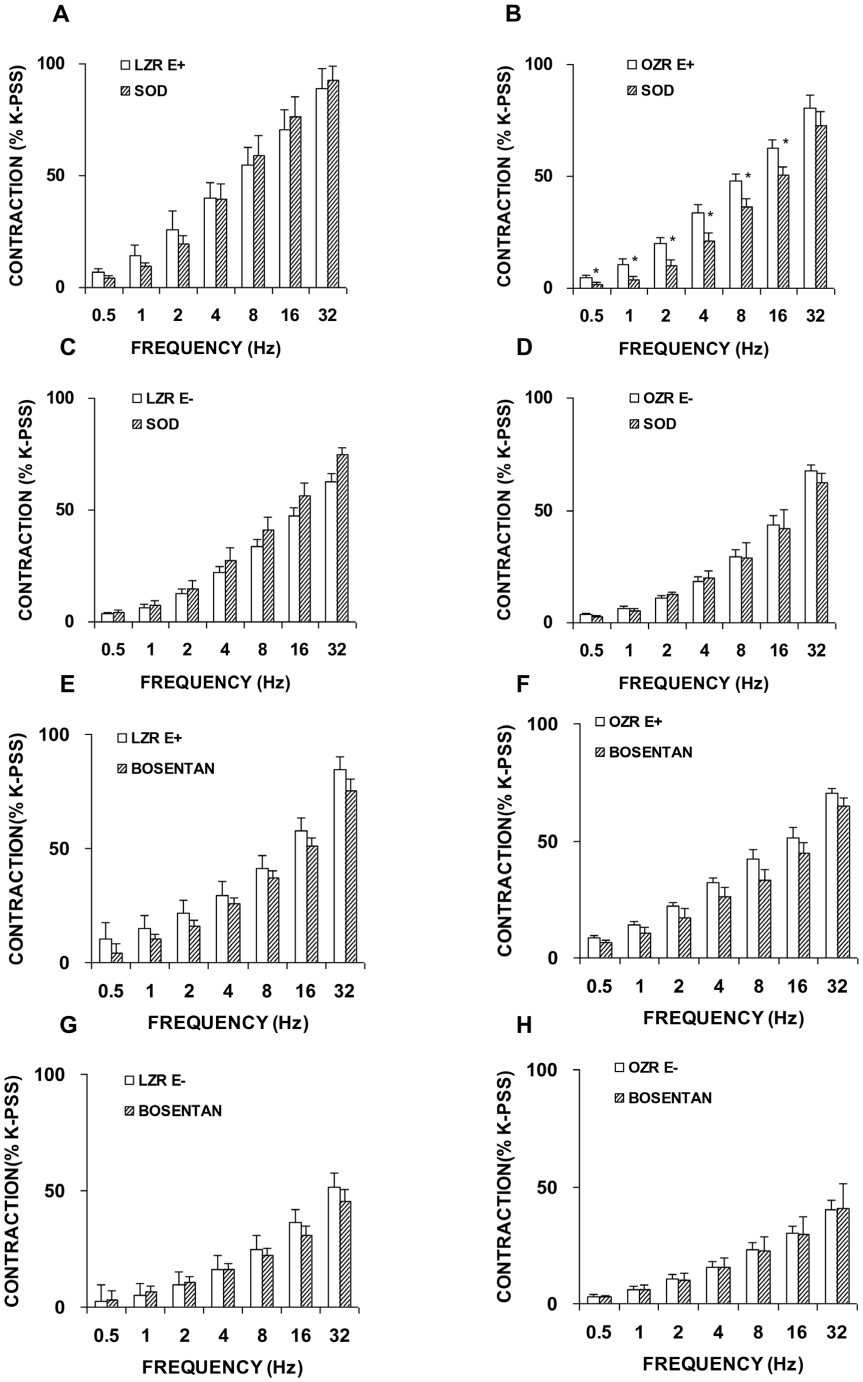
Other contractile factors involved in contractile responses induced by EFS. Neurogenic contractions were obtained in the absence and presence of an antioxidant enzyme, superoxide dismutase (SOD, 150 U/ml) in femoral artery with endothelium (E+) from LZR (A) and OZR (B), without endothelium (E−) from LZR (C) and OZR (D). Neurogenic contractions were obtained in the absence and presence of the non-specific endothelin (ET_A/B_) receptor blocker, bosentan (10 microM) in femoral artery with endothelium (E+) from LZR (E) and OZR (F), without endothelium (E−) from LZR (G) and OZR (G) segments. Data are means ± S.E.M. for values obtained in 6–7 animals. *P<0.05.

Independently of the presence of endothelium, the neurogenic response was unaltered by prior incubation with the superoxide-scavenging compound, SOD (150 U/mL), in segments from LZR (Figure 10A and C). However, SOD decreased EFS-induced contractions in endothelium-intact arteries from OZR (Figure 10B) but did not modify those produced in arteries without endothelium (Figure 10D).

EFS-contractile response curves were unmodified by the vascular NADP(H)-oxidase inhibitor apocynin (100 microM) in endothelium-intact and denuded femoral arteries from both groups (data not shown).

However, femoral arteries with or without endothelium from LZR and OZR showed a similar contraction response to EFS in the presence of the non-selective endothelin (ET_A/B_) receptor blocker, bosentan (10 microM) (Figure 10E–H).

## Discussion

The present study provides evidence of diminished neurogenic contractions due to endothelial dysfunction in femoral arterial preparations from OZR. In this model of obesity, the compensatory expression and functional involvement of nNOS and iNOS could be a protective strategy that generates superoxide anions in place of endothelial NO. At this stage, NO from non-eNOS could exert a considerable inhibitory effect that overrides the functional response of both augmented COX-2 expression coupled to vasoconstrictor release and enhanced ROS production, thus contributing to the reduced magnitude of neurogenic responses.

The OZR model has been well defined in terms of biochemistry and vascular function. We reported previously that OZR exhibits characteristics of the typical metabolic syndrome that accompanies prediabetes or insulin resistance in humans [11]. Oltman et al. [17] reported different rates of the development of vascular and neural dysfunction in diabetic and obese Zucker rats and attributed the difference to hyperglycemia, indicating that microvascular impairment preceded the appearance of nerve dysfunction [17]. The predominant nerve dysfunction mechanism observed in obesity of increased vasoconstrictor responses to EFS is driven by sympathetic nerve activation or direct adrenergic receptor stimulation [18], [19]. However, it is not clear whether there is an etiological mechanism in patients that is nerve-specific, rather than being simply a reflection of the neural consequences of the physiological challenge of vascular dysfunction. In the present study, obesity led to diminished neurogenic contractions, which could represent a mechanism designed to preserve adequate blood flow to the lower limbs in individuals in a prediabetic state. This reduced reactivity to constriction was not attributable to changes in the intrinsic contractile machinery as indicated by the similar depolarization -induced contractions elicited by a high potassium solution in LZR and OZR, as in the coronary arteries from this rat model. On the contrary, in penile arteries from OZR the reduced maximal contractile responses elicited by K-PSS probably reflect the smaller size of arteries due to structural remodelling [10]. The fact that guanethidine, phentolamine and prazosin greatly diminished the vasoconstriction response to EFS in arterial segments from both groups of rats, indicates that noradrenaline release from periarterial adrenergic nerves mediates this response by activating postsynaptic alpha_1_-adrenoceptors, as reported for the femoral artery of Wistar rats [20]. Thus, the differences in neurogenic contraction observed may not be attributed to release of noradrenaline and/or adrenergic innervation differences between LZR and OZR.

In contrast with our findings, adrenergic hyperactivity has been observed in a setting of obesity [18], [19]. However, these experiments were performed in endothelium-denuded segments and interactions between endothelium-derived factors and neurotransmitters may affect EFS responses [21].

Our observations suggest that endothelial dysfunction was responsible for the diminished contractions observed in OZR since both groups of animals showed a similar response when experiments were conducted on denuded segments. Other studies have also revealed endothelial dysfunction in penile arteries in the same model [10], [11].

It has been well established that endothelial mediators may indirectly regulate vascular function by modulating sympathetic neurotransmission [15], [21]–[23]. In fact, vasoconstriction resulting from the release of noradrenaline from sympathetic nerve terminals induces the release of NO from the endothelium [15], [21], [23].

Through immunohistochemical labelling, we localized eNOS at the endothelium and its expression detected by Western blotting was unmodified in our OZR. Upon nerve stimulation, NO synthesis inhibition by L-NOARG led to enhanced vasoconstriction of the intact femoral artery in both LZR and OZR. However, we detected NOS activity in denuded femoral arteries only in OZR. These findings point to modified eNOS activity in OZR and the similar L-NOARG effect observed in arterial rings with and without endothelium may be the outcome of inhibition of other NOS isoforms. Possible factors contributting to altered eNOS activity in OZR vessels include eNOS enzyme phosphorylation or other posttranslational modifications or the increased expression/activity of isoforms such as nNOS and iNOS. The latter is supported by observations that when NOS was blocked denuded femoral artery rings from OZR showed enhanced neurogenic vasoconstriction together with an increased response to EFS by the highly selective nNOS and iNOS inhibitors. Therefore nNOS and iNOS seems not to be activated in control conditions. The protein expression of both isoforms determined by immunohistochemical and biochemical procedures was higher in the femoral arteries from OZR. Evidence from several studies suggests that in mice genetically deficient in one of the constitutive NOS isoforms, the remaining constitutive isoform takes on some of the functions normally attributed to the deleted enzyme [24]. Under arteriosclerotic/inflammatory conditions accompanied by eNOS dysfunction, nNOS serves as an alternative source of NO, inhibiting vascular lesion formation and a rise in vascular tone [24]. Interestingly, in our study nNOS was detected both in the adventitial perivascular nerves and the endothelium lining in preparations from OZR. This observation is in agreement with earlier reports in which nNOS was detected in the neointima, endothelial cells and macrophages in both early and advanced atherosclerotic lesions [25]. The role of nNOS expressed in these vascular lesions remains to be determined.

In recent years, iNOS has been attributed an ever increasing role in mechanisms of vascular dysfunction, whereby the overproduction of NO has been linked to several pathophysiological conditions including insulin resistance and vascular inflammation [26]. Hence, in our femoral artery preparations from obese animals, the loss of endothelial NO was partially offset by activation of these alternative pathways, including the production of NO derived from nNOS and iNOS, which may avoid an increase in vascular tone.

In a previous study, we detected a dysfunctional nitrergic system in penile arteries from insulin resistant OZR that was able to explain enhanced noradrenergic vasoconstriction elicited by nerve stimulation [14]. These findings suggest that obesity may differentially impair neurogenic responses in different vascular settings.

Endothelial dysfunction has been described as the consequence of increased oxidative stressor production. Reactive oxygen species (ROS), mainly superoxide anions, are highly reactive and destroy NO, thus reducing its bioavailability and producing peroxynitrites. One source of ROS is uncoupled eNOS. eNOS uncoupling is a process whereby eNOS generates superoxide (O_2_^−^) in conditions of depleted substrate or NOS cofactor concentrations [27]. Elevated superoxide levels were detected in epineurial arterioles and aorta in obese rats developing microvascular and neural dysfunction [17].

Under these same conditions of insulin resistance, modified neural NO-mediated relaxant responses due to oxidative stress have been reported in penile arteries [14]. The present results suggest that oxidative stress may be involved in the changes that limit the activity of endothelial NO, due to the inhibitory effect of SOD in femoral arteries from obese animals with an intact endothelium but not in denuded preparations. This effect was not observed in the femoral artery from LZR. Thus, it is likely that endothelium- derived ROS production may explain the absence of L-NOARG effect noted in intact femoral artery segments from OZR.

Superoxide anions are generated by several sources including, besides uncoupled eNOS, NADPH oxidase and COX. Since the major source of ROS in the vasculature is vascular NADPH oxidase [28], it could be a key enzyme linked to oxidative stress in this pathogenic cascade. However, vascular NADPH oxidase was ruled out indicating the uncoupling of eNOS and/or COX as possible mechanisms responsible for the increased ROS levels observed here in femoral arteries from OZR.

In a setting of obesity, vasodilation in response to endothelium-derived hyperpolarization (EDH) may be impaired due to changes in underlying potassium channel signalling mechanisms. In obese rat saphenous artery, the role of NO was significantly impaired and offset by the appearance of IK_Ca_ and myoendothelial gap junctions, to maintain endothelium-dependent vasodilation [29]. The findings of our study suggest that the Na^+^-K^+^ ATPase pump, K_ATP_, K_ir_, IK_Ca_ and SK_Ca_ did not contribute to EFS-induced contractile responses in both strains of rats. In contrast, BK_Ca_ and K_V_ were identified here as K^+^ channels modulators of neural regulation in LZR femoral adrenergic transmission yet were unaffected by the conditions of obesity. As for the other components of the vasculature, during persistent high blood pressure, potassium channels in the plasma membrane of vascular smooth muscle cells undergo remodelling to maintain a heightened vascular tone [30]. In the present study, the preserved function of K^+^ channels in OZR could be explained by the lack of hypertension in our model [10], contrasting with the mild hypertension present in the diet-induced obesity rodent model [31].

There is mounting evidence to support the idea that the enhanced constrictor prostanoid production may play a key role in the pathogenesis of vascular dysfunction associated with diabetes. Cyclooxygenase enzymes may cause vascular hyper-contractility by increasing the synthesis of constrictor prostanoid(s) and excessive ROS formation [8], [32], [33].

In the vascular wall, both endothelial and smooth muscle cells contain COX-1 and COX-2. However, the endothelial cells of healthy blood vessels appear to contain much more COX-1 enzyme than surrounding smooth muscle cells [34]. Originally it was thought that COX-2 was largely responsible for the pathological production of prostanoids, for instance in inflammatory conditions, but it is now understood that COX-2 may play both a physiological and pathological roles [35].

The present results indicate that contractile prostanoids evoked by EFS in arteries from LZR are in fact derived from COX-1 and COX-2 in the endothelium, since non-selective and selective inhibitors of both isoforms reduced EFS-responses in endothelium-intact rings but did not alter contractions in denuded preparations. Moreover, the presence of COX-1 and COX-2 in femoral arteries from LZR detected by immunohistochemical staining and Western blotting, supports the fact that prostanoid synthesis could occur in the endothelium. Prostanoid derivative synthesis could explain the reduction observed in neurogenic contractions when comparing endothelial-intact with denuded femoral artery rings. Thus, activation of both COX isoforms could be an integral part of the endothelium-derived mechanisms that regulate local femoral vascular function in LZR, mainly via the formation of contractile prostanoids. This observation is consistent with the findings of a previous study in which we noted that in penile artery preparations from the same obese rat model, constitutive active COX-1 and COX-2 pathways were involved in regulating vascular tone in physiological conditions [12].

By immunostaining, COX-1 and COX-2 were also localized in the endothelium of femoral arteries from OZR. Further, COX-2 was also expressed in adventitial cells of arteries from OZR. While in obesity COX-1 expression remained stable, the greater expression of COX-2 protein was detected by Western blotting. Thus, it seems that when COX-1 enzyme is dysfunctional in OZR, COX-2 is able to metabolize arachidonic acid into vasoactive prostanoids [13]. The fact that the COX-2 inhibitor NS-398 reduced EFS-responses in femoral rings with or without endothelium from obese animals suggests that contractile prostanoids could also be produced from a non-endothelial source, for instance, perivascular macrophages. Thus, in OZR, the relaxant opposing effect of NO derived from neural and inducible sources probably hinders prostanoid-induced contraction leading to an EFS contractile response of lesser magnitude. Therefore, it is possible that these mediators, NOS and COX derivatives, interact with each other to maintain an appropriate basal tone. Oxidative stress could activate COX, which in turn, would give rise to the increased formation of vasoconstrictor prostanoids. These would also most likely drive the enhanced formation of ROS, leading to a vicious positive-feedback cycle of ROS production perpetuating the endothelial dysfunction [36]. Our findings point to excess ROS as a possible mechanism for the increased COX-2 expression observed in obese rats [34], [36].

In a prediabetic state in which compensating mechanisms predominate, it could be that the disruption of a COX pathway will trigger other metabolic pathways [37] contributing to other important physiopathological alterations, such as platelet aggregation or changes in the stability of atherosclerotic plaques [37], [38]. The outcome of COX-2 activity and subsequent prostanoid signalling will therefore depend on highly dynamic balance among these different and often conflicting signals. The impact of the vasoconstrictor effect of prostanoid synthesis may also increase as diabetes sets in [39].

The endothelium also produces endothelin-1 (ET-1), another essential factor for modulating vascular responses. In a setting of disease, this peptide has profound vasoconstrictor effects and contributes to changes in blood flow in the diabetic vasculature [40]. The results of the present study are inconsistent with the findings of studies that have indicated that overweight and obesity, independent of traditional cardiovascular risk factors, are associated with elevated ET-1- mediated vasoconstriction [41]. However, findings in this regard have been conflicting, with reports existing of either an increased or a normal contractile response to ET-1 in OZR arteries [42]–[44]. In fact, penile arteries harvested from the same rat model showed impaired ET-1 Ca^2+^ signalling along with changes in the ET receptor profile [45]. On the contrary, in the present study based on the lack of an effect of bosentan on EFS-induced contractions in LZR and OZR femoral rings, we were able to rule out ET-1 as the mediator involved in regulating contractile neurotransmission, reinforcing the idea that obesity does not equally affect all vascular beds.

The endothelial dysfunction observed here in femoral arteries from OZR may be attributable to diminished endothelial NO production leading to ROS. A compensation mechanism may exist to preserve relaxation involving NO derived from neural and inducible NOS to offset the EFS-evoked contraction when an essential vasodilator is lacking. Indeed, the augmented expression of nNOS and iNOS in OZR may serve as a protective strategy against the overexpression and contractile effect of COX-2. The present findings agree with the view that in most diseases there is a prodromal period in which the body tries to defend and maintain the organic homeostasis against an aggression (metabolic, infectious, etc.). In this case, obesity may precede a state of type II Diabetes Mellitus, the body defends itself against observed eNOS dysfunction and overexpression of COX-2 by activating iNOS and nNOS, as a compensatory mechanism, thus resulting in a sympathetic vasoconstriction response would be even lower than under control conditions. This compensatory mechanism gives a delayed onset of the clinical period of the cardiovascular disease. Our findings contribute to our understanding of interrelationships among NOS, COX and ROS. Future studies are needed to complete the picture of the cellular mechanisms of vascular dysfunction and unveil novel therapeutic targets to improve vascular health and reduce associated risks in obese individuals.
